# Cell Differentiation Trajectory-Associated Molecular Classification of Osteosarcoma

**DOI:** 10.3390/genes12111685

**Published:** 2021-10-23

**Authors:** Ankai Xu, Chao Qian, Jinti Lin, Wei Yu, Jiakang Jin, Bing Liu, Huimin Tao

**Affiliations:** 1 Department of Orthopedics Surgery, The Second Affiliated Hospital, Zhejiang University School of Medicine, No. 88, Jiefang Road, Hangzhou 310009, China; 21718246@zju.edu.cn (A.X.); med_qianchao@zju.edu.cn (C.Q.); 11918336@zju.edu.cn (J.L.); 21518152@zju.edu.cn (W.Y.); 21918384@zju.edu.cn (J.J.); liubingzju@zju.edu.cn (B.L.); 2 Orthopedics Research Institute of Zhejiang University, No. 88, Jiefang Road, Hangzhou 310009, China

**Keywords:** tumor/cancer stem cell, differentiation trajectory, molecular typing

## Abstract

This study aims to investigate the differentiation trajectory of osteosarcoma cells and to construct molecular subtypes with their respective characteristics and generate a multi-gene signature for predicting prognosis. Integrated single-cell RNA-sequencing (scRNA-seq) data, bulk RNA-seq data and microarray data from osteosarcoma samples were used for analysis. Via scRNA-seq data, time-related as well as differentiation-related genes were recognized as osteosarcoma tumor stem cell-related genes (OSCGs). In Gene Expression Omnibus (GEO) cohort, osteosarcoma patients were classified into two subtypes based on prognostic OSCGs and it was found that molecular typing successfully predicted overall survival, tumor microenvironment and immune infiltration status. Further, available drugs for influencing osteosarcoma via prognostic OSCGs were revealed. A 3-OSCG-based prognostic risk score signature was generated and by combining other clinic-pathological independent prognostic factor, stage at diagnosis, a nomogram was established to predict individual survival probability. In external independent TARGET cohort, the molecular types, the 3-gene signature as well as nomogram were validated. In conclusion, osteosarcoma cell differentiation occupies a crucial position in many facets, such as tumor prognosis and microenvironment, suggesting promising therapeutic targets for this disease.

## 1. Introduction

As the most common malignant primary bone tumor, it is reported that osteosarcoma often presented resistance to the methods of checkpoint blockade, such as inhibitors for PD-1/PD-L1 [1,2]. In addition, as the mainstay of therapy, chemotherapy is not always resultful. It is obvious that other efficient treatment thoughts are urgently needed. Naturally, tumor stem cell, as one of the reasons for immunologic escape and resistance of treatments, have emerged into researchers’ vision.

Tumor heterogeneity took part in many facets in the progression of cancer including therapy resistance, recurrence and metastasis [3]. The same as other many kinds of malignant tumors, osteosarcoma has been reported extensive intratumorally heterogeneity [4,5]. Tumor stem cell, or cancer stem cell, was defined those clones drive tumor initiation, also as a crucial contributor to tumor heterogeneity [6]. Although the conception of cancer stem cell is still controversial and often been confused with cancer initiating cells, it has been reported that there are some unique subsets of tumor clones with high-grade stemness traits as well as oncogenicity [7,8].

In osteosarcoma, researchers never ceased to explore the tumor heterogeneity and tumor stem cells with various methods. Using the suspension culture, Gibbs with his group obtained a small subset of self-renewing bone sarcoma cells with high expression of Oct 3/4 and Nanog [9]. Bulk RNA sequencing (RNA-seq) technology only provides us increasing knowledge of the osteosarcoma genome and the understanding of intra-tumor heterogeneity [7,10]. In previous study, multiregional sequencing was also an appropriate to explore heterogeneity in osteosarcoma [11]. Due to bulk RNA sequencing provides the average expression level of tissues, as the technology advances, single-cell RNA-seq (scRNA-seq) afford an opportunity to us that learn about tumor heterogeneity in the cellular level and explore tumor stem cells in greater depth [5].

In our study, using scRNA-seq data of primary osteosarcoma as well as trajectory analysis, time-related genes and cell differentiation-related genes were identified. Mutual genes were recognized as osteosarcoma tumor stem cell-related genes (OSCGs) for the next analysis. Next, we included osteosarcoma patients from Gene Expression Omnibus (GEO) database and obtained prognostic genes. Moreover, based on the expression patterns of prognostic OSCGs, in the GEO cohort, two OSCG-based molecular subtypes were identified and this cell differentiation state-based classification was proved prognostic correlative as well as tumor immunomicroenviroment related. Additionally, we explored the biological functions of prognostic OSCGs and as expected found that they are related to ossification, osteoblast differentiation and collagen metabolism. Then, latent available drugs were also revealed. Further, *CKLF*, *DKK1* and *MYC* were identified as the 3 key OSCGs which also effectual in bulk RNA sequencing, and the nomogram consisting of these 3 OSCGs and tumor stage at diagnosis was constructed. Finally, the above findings were validated using Therapeutically Applicable Research to Generate Effective Treatments (TARGET) osteosarcoma patient cohort. Brief intratumorally osteosarcoma cell differentiation states were discovered and the OSCGs played crucial roles in predicting the clinical outcomes as well as tumor immunomicroenviroment.

## 2. Materials and Methods

### 2.1. Data Sources and Data Preprocessing

The scRNA-seq data were obtained from the GSE152048 dataset in the GEO (https://www.ncbi.nlm.nih.gov/geo/query/acc.cgi?acc=GSE152048, accessed on 9 August 2021) database. 7 samples of primary osteosarcoma (BC2, BC3, BC5, BC6, BC16, BC21, BC22) were collected. Next, scRNA-seq data were processed with the Seurat package (version 3.1.5; http://satijalab.org/seurat/, accessed on 9 August 2021) in R software (version 3.6.1) for each individual sample [12]. Low-quality cells were excluded based on the following quality control standards: (1) genes detected in <200 cells were excluded; (2) cells with <1000 total detected genes were excluded; and (3) cells with ≥20% of mitochondria-expressed genes were excluded. After data filtering, using LogNormalize method as well as Harmony package (version 1.0), the data were normalized and removed the batch effects [13].

Further, we searched all the open samples with OS rate for osteosarcoma. Total three human datasets (GSE21257, GSE16091, GSE39055) in GEO (https://www.ncbi.nlm.nih.gov/geo/query/acc.cgi?acc=GSE21257; https://www.ncbi.nlm.nih.gov/geo/query/acc.cgi?acc=GSE16091; https://www.ncbi.nlm.nih.gov/geo/query/acc.cgi?acc=GSE39055, accessed on 9 August 2021) were collected to make GEO merged cohort. And the necessary documents were also respectively downloaded to annotate the respective probe sets into gene symbol sets. Finally, the “ComBat” algorithm was applied to reduce the likelihood of batch effects from non-biological technical biases [14].

And then samples including the prognosis information of osteosarcoma patients from TARGET (https://ocg.cancer.gov/programs/target/projects/osteosarcoma, accessed on 9 August 2021) were also gathered, and FPKM values were transformed into transcripts per kilobase million (TPM) values and then log2(TPM+1) values, which are more comparable between samples and more similar to data from microarrays [15,16]. All the gene matrices were standardized by quantiles normalization. Finally, 121 patients in GEO merged cohort and 95 patients in TARGET cohort were selected.

### 2.2. Dimensionality Reduction and Cell Annotation

Based on the integrated joint embedding produced by Harmony with the Louvain algorithm as well as the *t*-SNE algorithm, the dimension reduction of the scRNA-seq data was accomplished and the major clusters was obtained. Additionally, sub-clustering analysis was applied a similar procedure.

Using FindAllMarkers function in Seurat, differentially expressed genes (DEGs) between the cell clusters were gathered. The cell groups were annotated based on the previous study [5].

### 2.3. Pseudotime and Trajectory Analysis

Pseudotime and trajectory analyses of osteosarcoma cells were carried out by the ‘Monocle’ package (version 2.18.0) [17]. DEGs with *q*-value < 0.01 between the sub-cell groups were applied for dimension reduction with the reduceDimension function using the parameters reduction_method = “DDRTree” and max_components = 2. Based on plot_cell_trajectory function, osteosarcoma cells were ordered and visualized. Time-related genes were calculated (*q*-value < 0.01) and exhibited via plot_cell_trajectory and plot_pseudotime_heatmap function. BEAM analysis was performed (*q*-value < 0.01) to find cell differentiation-related genes visualized by plot_genes_branched_heatmap function.

### 2.4. OSCG-Based Molecular Subtypes of Osteosarcoma Patients

In GEO cohort, the Kaplan-Meier survival analysis based on the median of gene expression and univariate Cox hazard analysis were applied to screen OSCGs significantly associated with OS rate (*p* < 0.05).

In the discovery GEO and validation TARGET cohorts respectively, unsupervised consensus clustering was used to discover OSCG-based osteosarcoma subtypes [18]. In detail, a bootstrap procedure was performed with 80% item resampling 1000 times using the agglomerative kmeans clustering algorithm with the Euclidean distance metric. In the number of clusters from 2 to 9, the optimal number of clusters was selected that corresponds to both stable consensus matrices and unambiguous cluster assignments. Moreover, to validate the result, IGP analysis was used to evaluate the reproducibility of the clusters derived from consensus clustering in the two independent cohorts [19]. Due to the different types of expression values in the two datasets, we normalized the expression data by Z-score prior to the IGP statistical analysis.

### 2.5. Tumor Microenvironment Evaluation and Latent Drugs Related to Molecular Subtypes

Via ‘ESTIMATE’ package, the immune/stromal scores and tumor purity of each sample were calculated [20]. Tumor immune cell infiltration situation of 22 immune cells was identified by CIBERSORT algorithm [21]. Based on ‘limma’ package, the discrepancy of immune cells’ infiltration degree was evaluated, and a violin plot of immune cells with significant differences was presented. Using the Connectivity Map database (Previous version; https://portals.broadinstitute.org/cmap/, accessed on 9 August 2021), available drugs for influencing osteosarcoma OSCGs were revealed.

Additionally, GO analyses were realized via “clusterProfiler” package, and *p* adjust < 0.05 was believed to be statistically significant [22].

### 2.6. Risk Model Construction and Evaluation

In GEO cohort, we used the randomSurvivalForest algorithm to rank the importance of OSCGs [23]. We identified genes with a relative importance >0.50 as the final signature. Next, the multivariate Cox regression analysis was performed to build the risk score model and the model formula was showed above-mentioned.

Via timeROC package in R, ROC and AUC was performed to evaluate the classification efficiency of the prognosis predictions for 1 year, 2 years, 3 years and 5 years [24]. Kaplan-Meier survival analysis was performed to estimate the OS of two groups, and survival differences were evaluated by a two-sided log-rank test.

### 2.7. The Construction of Nomogram

Univariate and multivariate Cox regression analyses were performed in both osteosarcoma cohorts to determine whether the predictive performance of risk scores could be independent of the clinicopathological variables. These variables included gender, age, tumor site, stage at diagnosis, stromal scores, immune scores, ESTIMATE scores, tumor purity and risk scores.

Via the “rms” package in R, independent predictors, metastasis stage at diagnosis and risk score were used to construct the nomogram. The predictive accuracy of the OSCG-based prognostic model was evaluated by Harrell’s concordance index (C-index). In addition, calibration curves were drawn to assess the consistency between actual and predicted survival.

## 3. Results

### 3.1. Identification of 8 Cell Clusters in Primary Osteosarcoma Using scRNA-seq Data

The schematic diagram of this study is illuminated in Supplementary Figure S1. From GEO database, seven raw output data of scRNA-seq for primary osteosarcoma were obtained.

**Figure 1:**
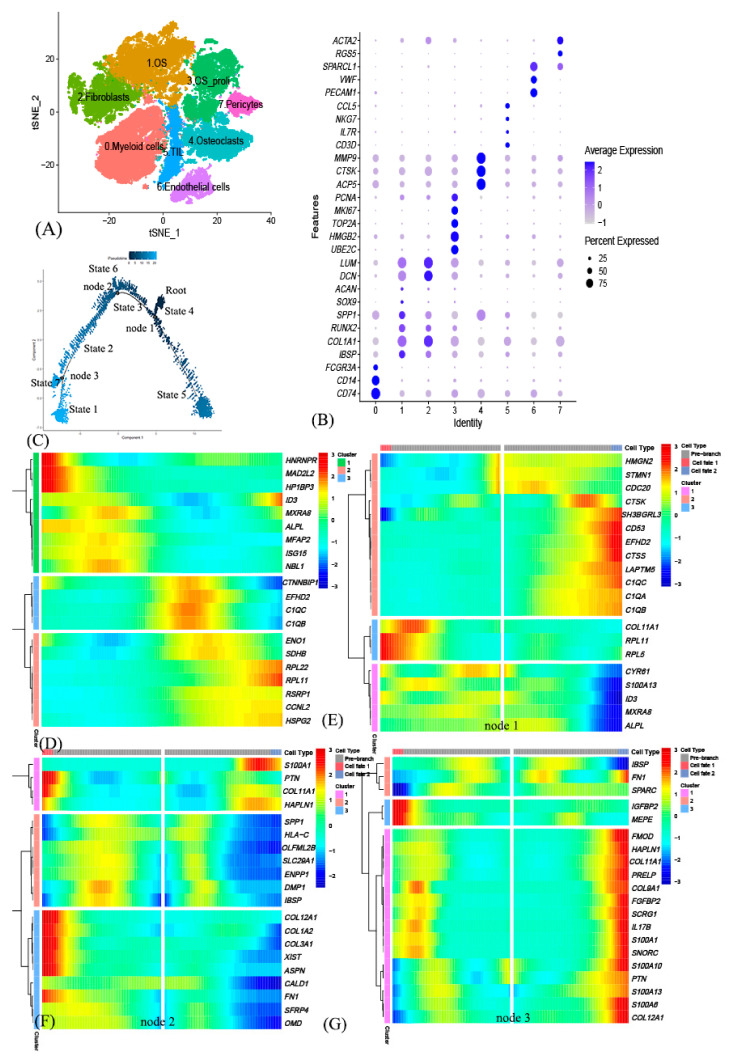
Single-cell transcriptomic analysis of osteosarcoma. (**A**) The t-distributed stochastic neighbor embedding (t-SNE) plot of the 8 identified main cell types. (**B**) Typical 28 signature gene expressions across cellular clusters. (**C**) The Monocle 2 trajectory plot showing the pseudotime curve of osteosarcoma subclusters. (**D**) Heatmap of the top 20 genes that were differentially expressed along the pseudotime. (**E**–**G**) Heatmap of the top 20 genes that were differentially expressed in each cell fate branch.

After initial quality control assessment and batch effects removal, we obtained single-cell transcriptomes from 67,146 cells for the further analysis. Based on the *t*-distributed stochastic neighbor embedding (*t*-SNE) algorithm, the cells in human osteosarcoma were successfully classified into 8 clusters (Figure 1A). And the typical canonical markers for major cell types were illuminated in Figure 1B (Supplementary Figures S2 and S3, Figure S4A), according to the original previous research [5]. Cluster 0, containing 15,420 cells, was annotated as myeloid cells; Cluster 1, containing 14,882 cells, was annotated as osteosarcoma cells; Cluster 2, containing 9528 cells, was annotated as fibroblasts; Cluster 3, containing 8876 cells, was annotated as osteosarcoma cells with high proliferation; Cluster 4, containing 8125 cells, was annotated as osteoclasts; Cluster 5, containing 4640 cells, was annotated as tumor infiltrating lymphocytes (TILs); Cluster 6, containing 3191 cells, was annotated as endothelial cells; Cluster 7, containing 2484 cells, was annotated as pericytes.

### 3.2. Cell Trajectory Analysis Identified OSCGs

Then, osteosarcoma subsets, Cluster 1 and Cluster 3, including 11,780 cells were extracted for further analysis (Supplementary Figure S4B–D). Trajectory analysis of osteosarcoma cells was performed based on the Monocle 2 algorithm, finding three differentiation nodes (Figure 1C) [17]. And then 859 pseudo time-related genes were obtained (Figure 1D). Via the branch expression analysis modeling (BEAM) analysis, three cell differentiation nodes-related genes were received respectively, and a total of 1042 genes were received. (Figure 1E–G). Combining with pseudotime-related genes and cell differentiation nodes-related genes, there were total 542 genes recognized as OSCGs (Supplementary Table S1).

### 3.3. The Prognostic OSCG-Based Molecular Subtypes of Osteosarcoma Patients

**Figure 2:**
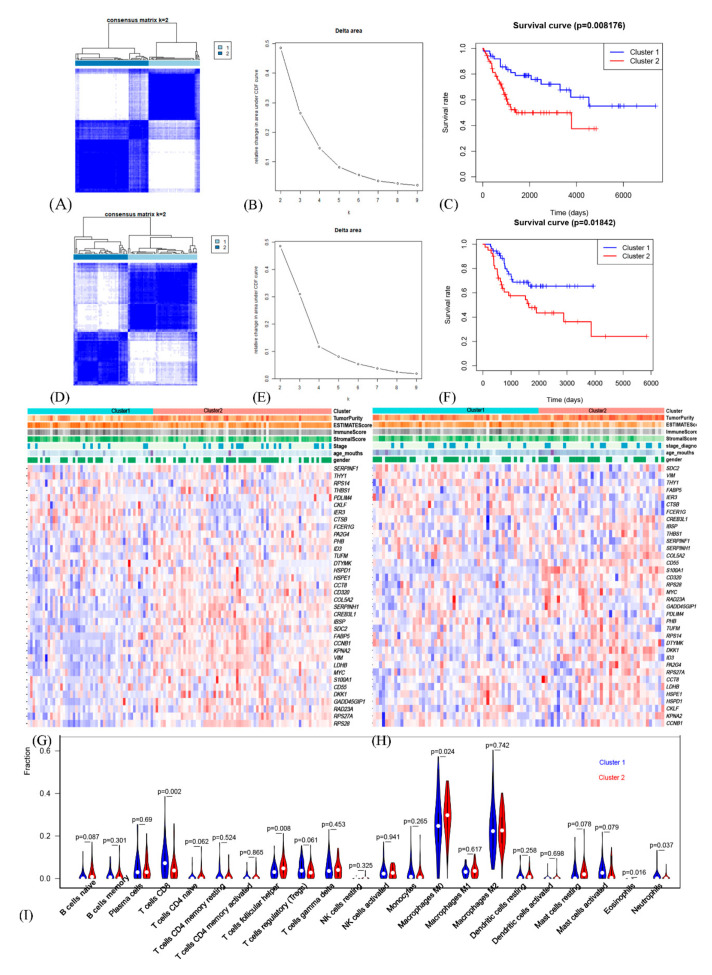
The prognostic OSCG-based molecular subtypes of osteosarcoma. (**A**) Consensus matrix heatmaps for the chosen optimal cluster number (k = 2) for the GEO cohort. (**B**) Cumulative distribution function (CDF) curves for the GEO cohort. (**C**) Kaplan-Meier analysis of OSCG-based molecular subtypes of osteosarcoma for GEO cohort. (**D**–**F**) The similar analyses in TARGET cohort. (**G**) Heatmap of prognostic OSCG-based genes expression, molecular subclusters, tumor microenvironment evaluation and clinicopathological characteristics in GEO cohort. (**H**) The similar heatmap in TARGET cohort. (**I**) The tumor immunocytes infiltration microenvironment across the two subtypes.

First, respectively, Kaplan-Meier survival analysis based on the median of gene expression and univariate Cox hazard analysis were applied to the GEO cohort to screen OSCGs significantly associated with overall survival (OS) rate. Subsequently, 36 prognostic OSCGs were obtained (Supplementary Table S2). Based on prognostic OSCGs and unsupervised consensus clustering analysis in the GEO cohort, two molecular subtypes of osteosarcoma samples were identified (Figure 2A) [18]. The 2-cluster solution corresponded to the largest cluster number that induced the least incremental change in the area under the cumulative distribution function (CDF) curves while keeping the maximal consensus within clusters and the minimal rate of ambiguity in cluster assignments (Figure 2B). Then, the Kaplan-Meier analysis determined the statistical significance of consensus clustering result of osteosarcoma that subtype_1 having better OS than subtype_2 (Figure 2C, *p* = 0.008176). Further, the same results were obtained in an external independent TARGET cohort (Figure 2D–F, *p* = 0.01842). Finally, the reproducibility of the two osteosarcoma subtypes across the discovery and validation cohorts was evaluated by in-group proportion (IGP) statistic [19]. The IGP values are 77.3 and 71.2% for subtype_1 and subtype_2, respectively, indicating that both subtypes had high consistency between the two cohorts.

### 3.4. Comprehensive Analysis of Tumor Microenvironment Scores and Immune Cell Infiltration across Molecular Subtypes

According to tumor microenvironment evaluation, in GEO cohort, our study found that cluster_2 had significantly higher tumor purity (*p* = 0.00026) but lower ESTIMATE score (*p* = 0.00026), immune score (*p* = 0.000016) and stromal score (*p* = 0.012) than cluster_1 (Figure 2G, Supplementary Table S3) [20]. Additionally, there were no significant differences in age, gender and stage at diagnosis between two subtypes. Also, the same situation could be found in TARGET cohort, that cluster_2 had significantly higher tumor purity (*p* = 0.0012) but lower ESTIMATE score (*p* = 0.0012), immune score (*p* = 0.019) and stromal score (*p* = 0.00056) than cluster_1 and there were also no significant differences in age, gender and stage at diagnosis between two subtypes. (Figure 2H, Supplementary Table S3). Further, via the CIBERSORT algorithm, the tumor immunocytes infiltration microenvironment was evaluated (Figure 2I) [25]. Differential analysis found that cluster_2 contained more follicular helper T cells (*p* = 0.008) and M0 macrophages (*p* = 0.024), but less CD8 T cells (*p* = 0.002), implying the important function of immunotherapy as well as tumor-associated macrophages and suggesting the potential relationship between immunity and tumor stem cell.

**Figure 3:**
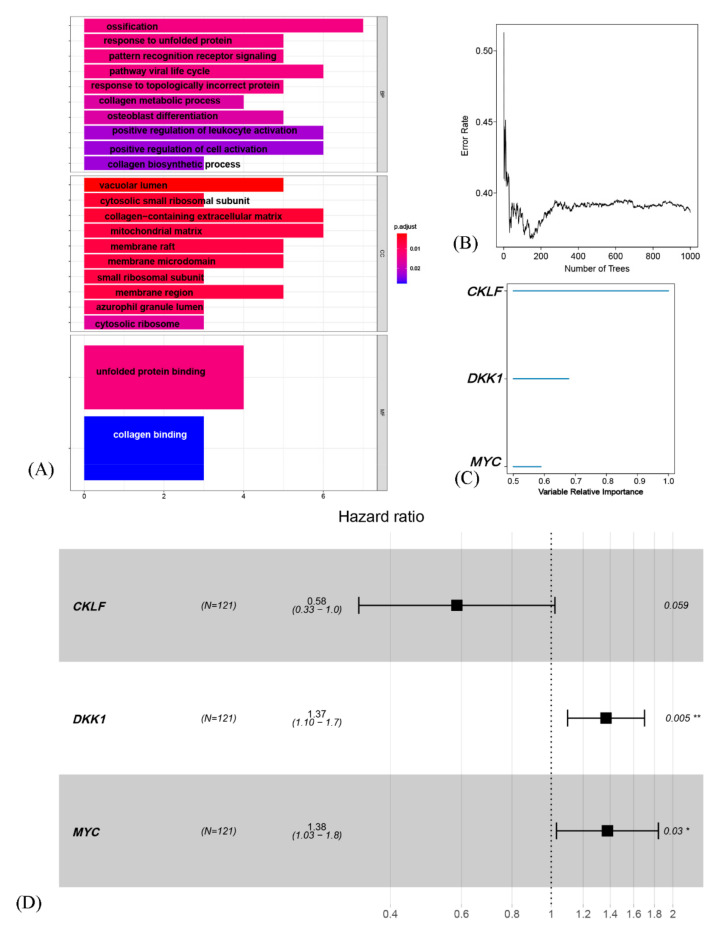
Generation a prognostic risk scoring signature (**A**) GO terms analysis for prognostic OSCGs. (**B**) Error rate for the data as a function of the classification tree. (**C**) out-of-bag importance values for the predictors. (**D**) Forest plot showing hazard ratio of the three genes via multivariate cox regression analysis in GEO. * *p* < 0.05, ** *p* < 0.01.

Additionally, using Gene Ontology analysis for prognostic OSCGs, the process of ossification, response to unfolded protein, response to topologically incorrect protein, osteoblast differentiation and collagen metabolism could have close relationship with cell differentiation trajectory of tumor stem cell in osteosarcoma (Figure 3A). These results supported that osteosarcoma are derived from progenitor cells in the osteoblast lineage [26,27]. Moreover, consistent with previous studies, osteoblast differentiation and the formation of osteospheres could reflect the stemness of osteosarcoma [28,29]. Although collagen metabolism is significant for mesenchyma stem cells, few studies have explored the relationship between collagen metabolism and osteosarcoma stem cells. Using the Connectivity Map database, available drugs for influencing osteosarcoma OSCGs were revealed, including proglumide, midecamycin, naproxen, ethambutol, denatonium benzoate, tiapride, salsolidin and pyrimethamine (Supplementary Table S4) [30].

### 3.5. Generation and Validation of a Prognostic Risk Scoring Signature

In GEO cohort, univariate Cox hazard analysis was performed with *p* < 0.05 as the threshold. Then, using the randomForestSRC software package, we ranked the importance of prognostic-OSCGs. The final signature was identified as genes with a relative importance >0.50 (Supplementary Table S5). The relationship between the error rate and the number of classification trees as well as the out-of-bag importance of the first three genes were then exhibited (Figure 3B,C).

Three genes (*CKLF*, *DKK1*, *MYC*) were identified from the random forest algorithm and subsequently multivariate Cox regression analysis were exerted to establish the 3-gene signature (Figure 3D). The model was as follows: RiskScore = −0.537343156 ∗ exp*CKLF* + 0.312512732 ∗ exp*DKK1* + 0.320496576 ∗ exp *MYC*.

**Figure 4:**
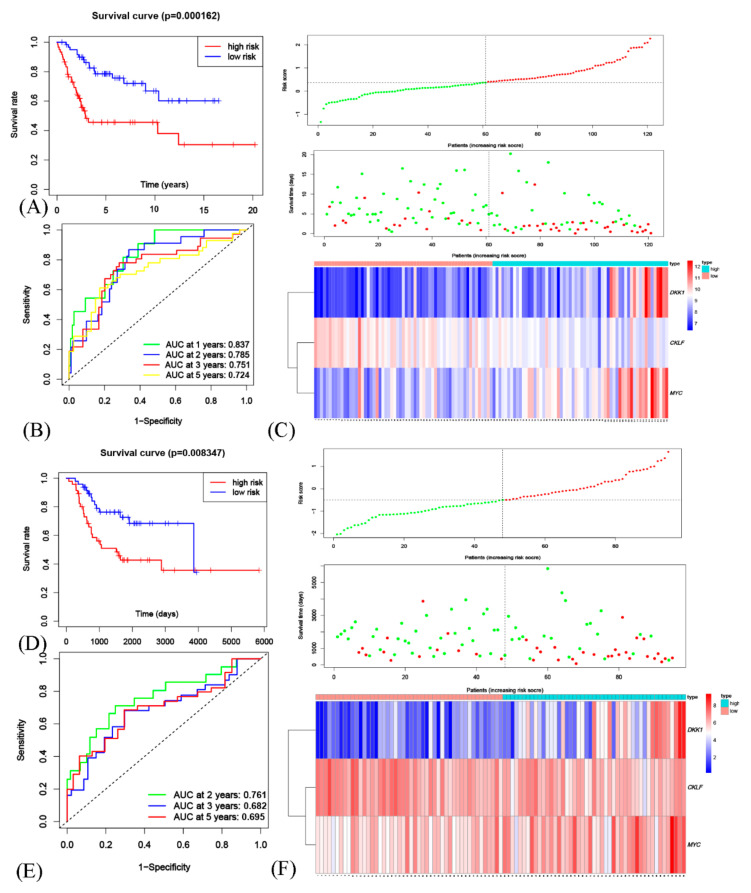
Validation of the OSCG-based risk score model in osteosarcoma patients. (**A**) Kaplan-Meier survival analysis to estimate the OS of high-risk and low-risk patients in GEO cohort. (**B**) Time-dependent ROC curve analysis was performed to evaluate the prognostic performance of the OSCG signature for predicting the 1-, 2-, 3- and 5-year OS rates in GEO cohort. (**C**) Risk score in the GEO cohort were calculated, and the patients were divided into either a high-risk group or a low-risk group using the median value. Risk sores, patient survival status and the signatures expression across the two groups were displayed in GEO cohort. (**D**–**F**) Similar analyses were performed in TARGET cohort using the median value as cutoff value.

In GEO cohort, the Kaplan-Meier curves were drawn (Figure 4A), and a markedly significant difference of *p* < 0.001 was observed between the groups by median cut-off value (Median cut-off value = 0.37). By the time-dependent receiver operating characteristic (ROC) curves, the area under the ROC curve (AUC) for 1-year, 2-year, 3-year, and 5-year OS were gathered (Figure 4B). Additionally, the distribution of risk scores in patients and the relationship between risk scores and survival time were displayed (Figure 4C). By heatmap, gene expression profiles in high-risk and low-risk were exhibited (Figure 4C). The genes with HR > 1(*DKK1*, *MYC*) were considered dangerous, while the gene with HR < 1 (*CKLF*) protective. Additionally, the high-risk group has the higher expression dangerous genes with the lower protective one.

Further, the 3-gene signature was validated in the external independent TARGET cohort. In TARGET cohort, by the median value as cut-off value, the Kaplan-Meier curves were drawn (Figure 4D), and a markedly significant difference of *p* < 0.01 was observed. By ROC curves, the AUC for 2-year, 3-year, and 5-year OS were gathered (Figure 4E). The gene expression heatmap, the distribution of risk scores in patients and the relationship between risk scores and survival time were also displayed (Figure 4F).

### 3.6. The Risk Model Based on OSCGs as an Independent Prognostic Factor

In GEO merged cohort, both univariate and multivariate analysis showed that the risk scores an independent prognostic indicator (HR = 2.718, 95%CI = 1.730–4.271, *p* < 0.001; HR = 3.148, 95%CI = 1.722–5.756, *p* < 0.001) (Supplementary Table S6). Further, we validated this model in the external independent TARGET cohort with the same way. And the risk score was still proved an independent prognostic factor the same as tumor stage at diagnosis (HR = 2.308, 95%CI = 1.498–3.554, *p* < 0.001; HR = 2.344, 95%CI = 1.419–3.870, *p* < 0.001).

### 3.7. The Construction of Nomogram for Predicting Patient 3-Year and 5-Year OS

**Figure 5:**
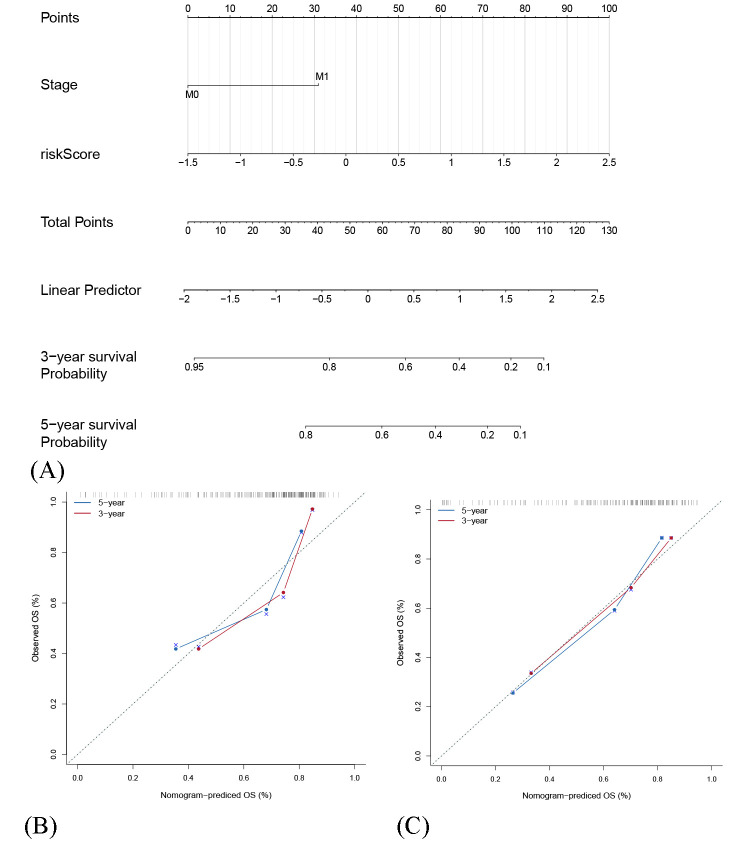
Construction and evaluation of a nomogram. (**A**) A nomogram for predicting 3-year and 5-year OS. (**B**) The calibration curves for predicting 3-year and 5-year OS in GEO cohort. (**C**) The calibration curves for predicting 3-year and 5-year OS in TARGET cohort.

In the clinical environment, a nomogram is a highly legible illustration of a mathematical model and a convenient tool for predicting the outcome of individual patients. The above analysis showed that the risk score as well as tumor stage at diagnosis were independent prognostic indicators. And in this study, the nomogram consisting of above significant risk factors was established, with C-index of 0.757 in GEO merged cohort and 0.773 in TARGET cohort (Figure 5A). The calibration curves showed non-significant deviations between predicted and actual probability in both GEO merged cohort and TARGET cohort (Figure 5B,C).

## 4. Discussion

Whereas the researches of the earlier years had noticed the existence of tumor stem cells (TSCs, also cancer stem cells—CSCs), the precise definition and the standard of perfection for TSCs are still deficient. Both tumor-initiating cells and drug-resistant cells could be deemed to TSCs. Similarly, cells with highly proliferation ability or cells in the dormancy stage may also be thought to TSCs [31].

In terms of TSCs in osteosarcoma, the scientists never ceased from exploration. There are many methods for obtaining TSCs: dye exclusion assays, utilizing aldehyde dehydrogenase, utilizing cell surface markers (such as *CD133*, *CD117*, *IBSP* and *CD105*) and sphere formation assays [8]. However, it is hard to identify quiescent TSCs. Moreover, the alleged TSC phenotypes may vary with diverse isolation methods [8]. With continuing development of scRNA-seq technology as well as machine learning, new methods as well as perspectives have been provided to us for seeking the OSCGs. In our study, 36 prognostic OSCGs were obtained to generate molecular subtypes of osteosarcoma. Among them, many genes have been proved related to cancer stemness and tumorigenesis in osteosarcoma. It has been reported that osteosarcoma may be origin from the mesenchymal stem cell (MSC) or the osteoblast [32]. In addition, transformed human MSCs or osteoblasts were observed acquiring malignant osteosarcoma-like properties [33,34,35]. From MSCs to osteosarcoma, *CDKN2A*, *ALPL*, *SPARC* (osteonectin) and *MYC* were considered as origin-related factors [32,35,36]. From osteoblasts to osteosarcoma, *SOX9*, *SPP1* (osteopontin) and *RUNX2* were also considered as origin-related factors [32,37]. Moreover, in patients with Li-Fraumeni syndrome, *DCN* (decorin) mediated Li-Fraumeni syndrome related osteosarcoma [33]. By the way, *CD117*, *IBSP* (Stro-1), *ID1*, *SOX9*, *CD24*, *CD44* and *THBS-1* were also reported to affect osteosarcoma growth and stemness [37,38,39,40,41,42].

Previous studies have indicated that some TSCs-related genes correlate with the prognosis for many neoplasms [43,44]. Moreover, due to the TSCs-related genes are not universal, specific researches should be implemented respectively for various neoplasms. Currently, aiming at TSCs-related hedgehog signaling pathway, vismodegib and sonidegib are approved by the US Food and Drug Administration (FDA) for use in advanced basal cell carcinoma [45]. In addition, *CD47*, was not only considered as TSCs-related gene in leukemia and bladder tumor, but also an important marker to evade phagocytosis [46,47]. Further, in clinical trials, *CD47* blockade combing rituximab in non-Hodgkin’s lymphoma showed promising activity [48]. In osteosarcoma, *Dkk1* was considered as molecule with pro-tumor function [49]. It is interesting that the clinical trials have already been in progress for *DKK1* inhibited drugs BHQ880 and DKN-01 in other tumors [50,51].

Although earlier studies mentioned above indicated the importance of TSCs in osteosarcoma, little attention was paid to using scRNA-seq technology for exploring TSC related genes neither related molecular subtyping. On the one hand, the heterogeneity of tumor makes it hard to target all the tumor cells. On the other hand, similar to traditional pathology types, molecular phenotype identification can also be used to optimize and simplify treatment strategies. Based on TSCs to construct molecular subtypes can obviously an appropriate strategy. Considering the TSCs may only occupy a small proportion of the total tumor cells, directly targeting well-known stem-cell markers like *CD133* may serve a restricted role. In our study, combining scRNA-seq data and bulk RNA-seq data develops better strategy. TSC related genes as well as molecular subtypes were obtained. Moreover, the molecular subtypes successfully predicted overall survival, tumor microenvironment and immune infiltration status, no matter the metastasis stage at diagnosis. Additionally, related latent drugs were exploited. Further, for the osteosarcoma population, our 3-gene risk score model based on OSCGs was shown to be a reliable predictor of prognosis. Subsequently, the nomogram consisting of significant risk factors was established.

However, the present study still has some limitations. Firstly, the race and district of patient were heterogeneous because of the retrospective multiple-platform study. Secondly, although gene symbols with changed official name were converted into latest edition as far as possible, some old gene symbols have been officially abandoned. Thirdly, integrated clinical characteristic was incomplete for osteosarcoma cohort, such as, the incomplete EFS or PFS curves, needing preferable clinical follow-up and data collection. Additionally, drug-resistant TSCs or dormancy stage TSCs were still hard to identify, needing more information involved. Finally, experimental studies as well as further prospective clinical studies are required to further validate and polish our results.

## 5. Conclusions

Via scRNA-seq data, bulk RNA-seq data and microarray data, we found that osteosarcoma samples could be briefly divided into two molecular subtypes associated with prognostic OSCGs. Moreover, the classification of osteosarcoma patients based on aforesaid patterns can to some extent predict patient overall survival, tumor microenvironment and immune infiltration status. Further, the key prognosis-predicting OSCGs were identified and a related nomogram was established. In summary, this study highlights not only the promise of researches about osteosarcoma cell differentiation and osteosarcoma stem cells, but the essential roles of corresponding molecular subtypes or OSCGs in predicting the clinical outcome and immune-micro-enviroment.

## Data Availability

Publicly available datasets were analyzed in this study. The data are accessible at the following repositories: https://www.ncbi.nlm.nih.gov/gds, https://ocg.cancer.gov/programs/target/data-matrix; accessed on 9 August 2021.

## Supplementary Materials

The following are available online at https://www.mdpi.com/article/10.3390/genes12111685/s1, Table S1: 542 tumor stem cell-related genes, Table S2: 36 prognostic tumor stem cell-related genes, Table S3: The differences between two subtypes via unsupervised clustering, Table S4: The latent drugs via prognostic tumor stem cell-related genes in osteosarcoma, Table S5: The top 10 outcome by random forest analysis, Table S6: To find independent predictors by univariate and multivariate cox regression analyses, Figure S1: Schematic diagram showing the study design and principal findings, Figures S2 and S3: Other marker gene expressions across cellular clusters, Figure S4: Single-cell transcriptomic analyses of all cells and the subclusters of osteosarcoma cells.
