# Supplementary material for: Cell Differentiation Trajectory-Associated Molecular Classification of Osteosarcoma
Source: Genes (Basel). 2021 Oct 23;12(11):1685. doi: 10.3390/genes12111685 (PMC8625454; doi:10.3390/genes12111685)
Supplement: Supplementary file 1 [file genes-12-01685-s001.zip › Supplement Figure S4.pdf]

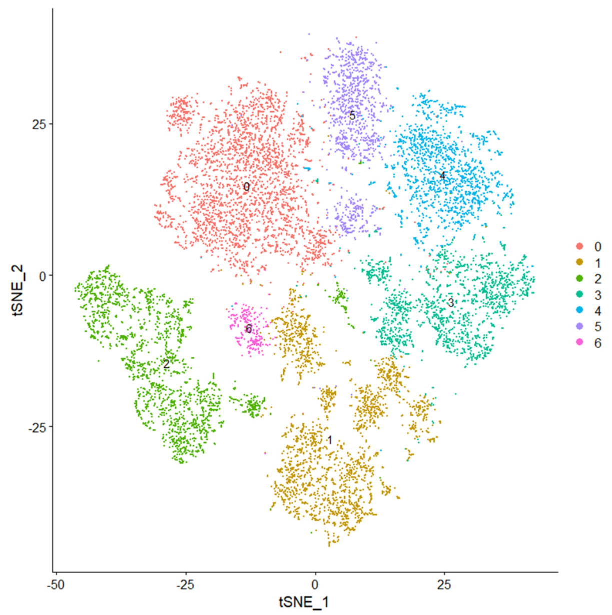

(A)

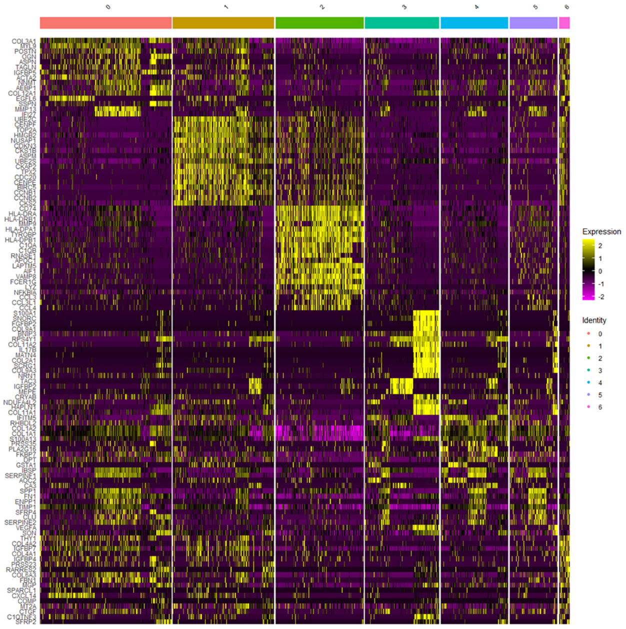

(B)

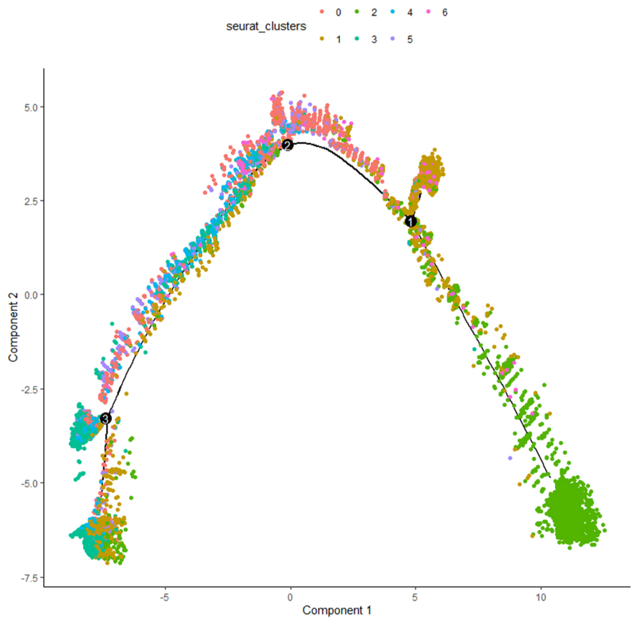

(C)

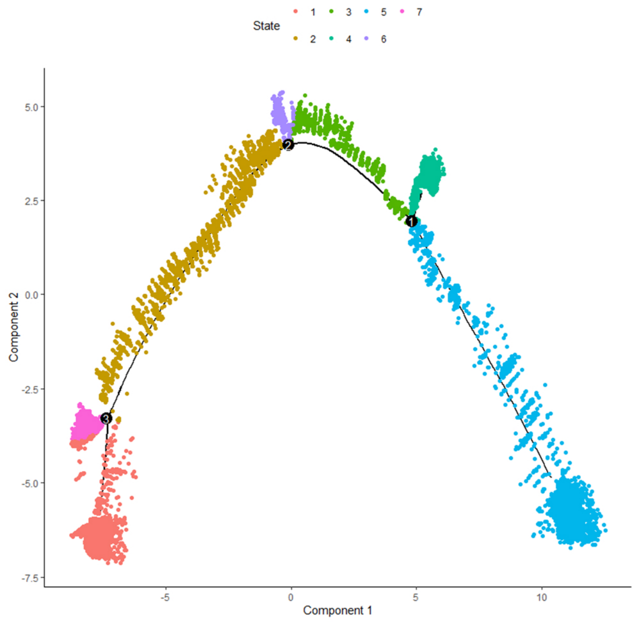

(D)

Supplement Figure S4: Single-cell transcriptomic analyses of all cells and the subclusters of osteosarcoma cells. (A) The top 5 marker genes of each subtype of all cells are displayed in the heatmap. (B) The t-distributed stochastic neighbor embedding (t-SNE) plot of the subtypes of osteosarcoma cells. (C) Via cell trajectory analysis, the top 5 marker genes of each state of osteosarcoma cells are displayed in the heatmap. (D) Pseudotime and trajectory analysis.
