# Supplementary figures and images for: Cell Differentiation Trajectory-Associated Molecular Classification of Osteosarcoma
Source: Genes (Basel). 2021 Oct 23;12(11):1685. doi: 10.3390/genes12111685 (PMC8625454; doi:10.3390/genes12111685)

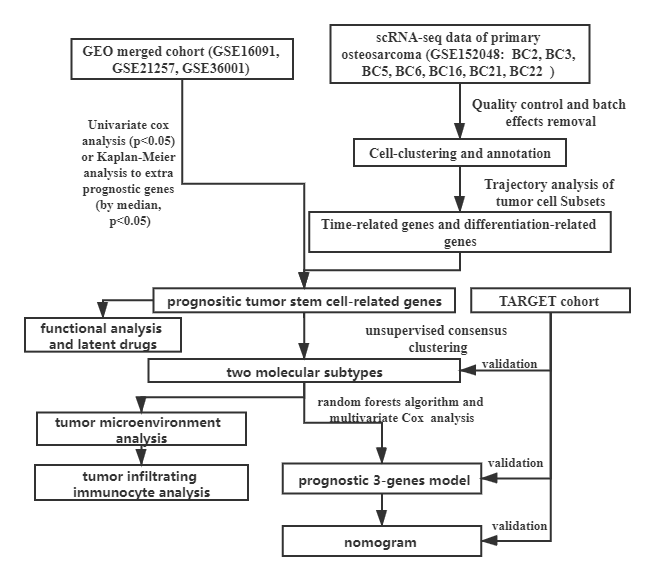

Supplement: Supplementary file 1 [file genes-12-01685-s001.zip › Supplement Figure S1.png]

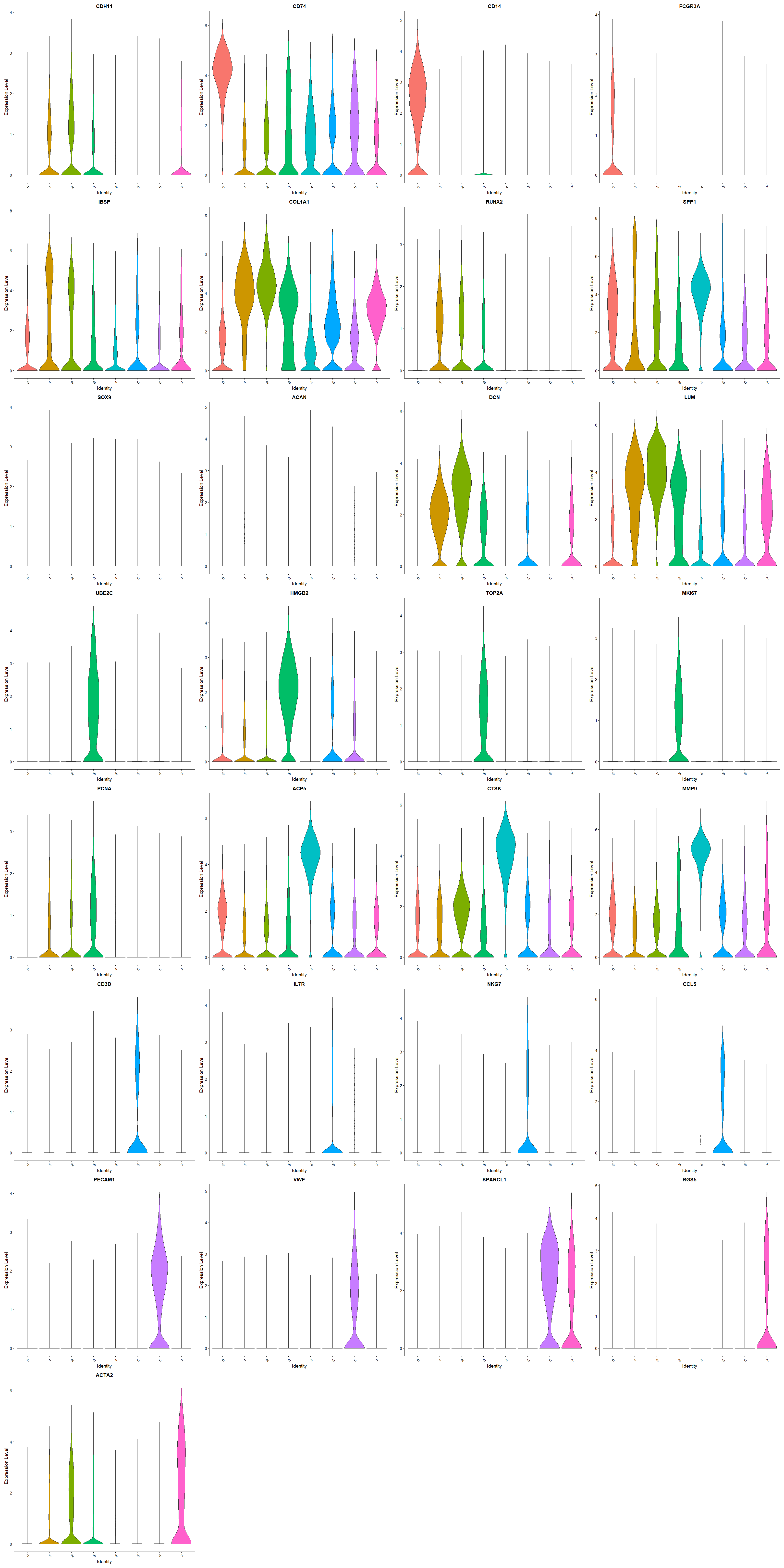

Supplement: Supplementary file 1 [file genes-12-01685-s001.zip › Supplement Figure S2.png]

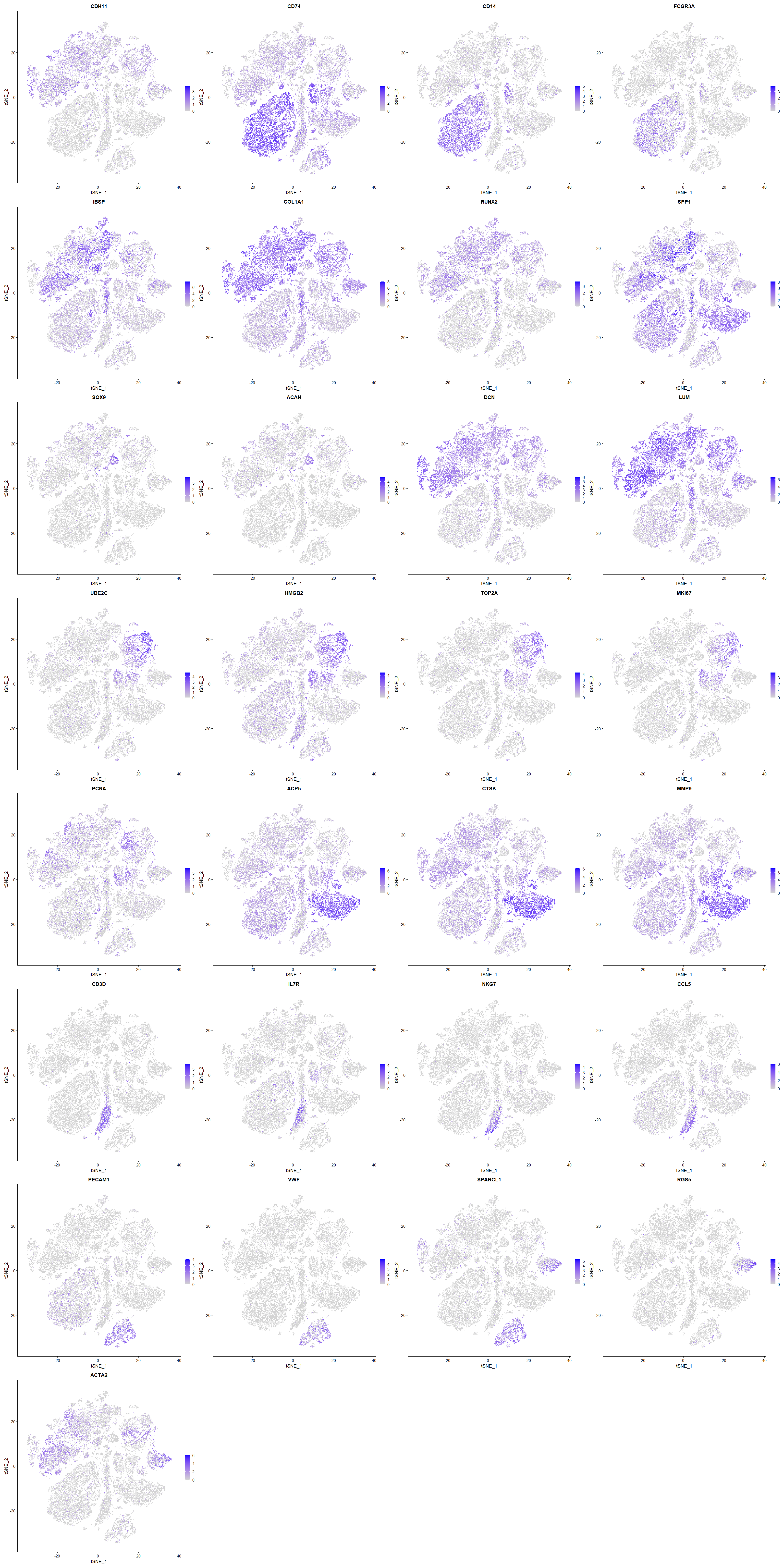

Supplement: Supplementary file 1 [file genes-12-01685-s001.zip › Supplement Figure S3.png]
